# Contact Resistance Engineering in WS_2_-Based FET with MoS_2_ Under-Contact Interlayer: A Statistical Approach

**DOI:** 10.1021/acsami.4c09688

**Published:** 2024-08-26

**Authors:** Małgorzata Giza, Michał Świniarski, Arkadiusz P. Gertych, Karolina Czerniak-Łosiewicz, Maciej Rogala, Paweł J. Kowalczyk, Mariusz Zdrojek

**Affiliations:** † Faculty of Physics, Warsaw University of Technology, Koszykowa 75, 00-662 Warsaw, Poland; ‡ Faculty of Physics and Applied Informatics, University of Łódź, Pomorska 149/153, 90-236 Łódź, Poland

**Keywords:** contact resistance, van der Waals heterostructure, interlayer, transition metal dichalcogenides, field-effect transistor, gold-assisted transfer

## Abstract

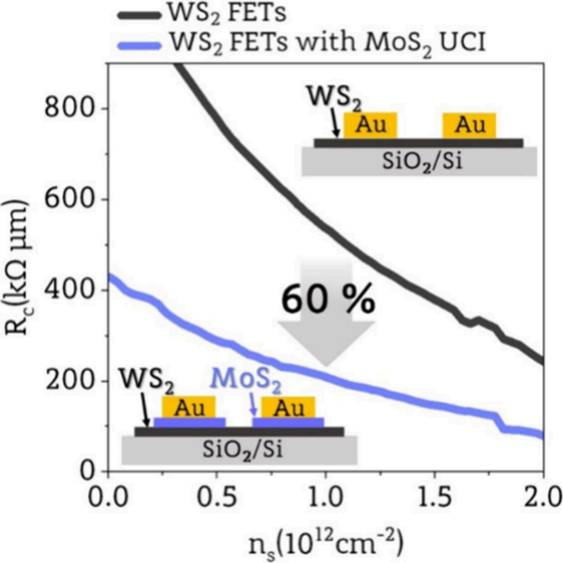

One of the primary factors hindering the development of 2D material-based devices is the difficulty of overcoming fabrication processes, which pose a challenge in achieving low-resistance contacts. Widely used metal deposition methods lead to unfavorable Fermi level pinning effect (FLP), which prevents control over the Schottky barrier height at the metal/2D material junction. We propose to harness the FLP effect to lower contact resistance in field-effect transistors (FETs) by using an additional 2D interlayer at the conducting channel and metallic contact interface (under-contact interlayer). To do so, we developed a new approach using the gold-assisted transfer method, which enables the fabrication of heterostructures consisting of TMDs monolayers with complex shapes, prepatterned using e-beam lithography, with lateral dimensions even down to 100 nm. We designed and demonstrated tungsten disulfide (WS_2_) monolayer-based devices in which the molybdenum disulfide (MoS_2_) monolayer is placed only in the contact area of the FET, creating an Au/MoS_2_/WS_2_ junction, which effectively reduces contact resistance by over 60% and improves the *I*_on_/*I*_off_ ratio 10 times in comparison to WS_2_-based devices without MoS_2_ under-contact interlayer. The enhancement in the device operation arises from the FLP effect occurring only at the interface between the metal and the first layer of the MoS_2_/WS_2_ heterostructure. This results in favorable band alignment, which enhances the current flow through the junction. To ensure the reproducibility of our devices, we systematically analyzed 160 FET devices fabricated with under-contact interlayer and without it. Statistical analysis shows a consistent improvement in the operation of the device and reveals the impact of contact resistance on key FET performance indicators.

## Introduction

Transition metal dichalcogenides (TMDs) are a broad family of materials with intriguing properties, making them highly attractive for a wide range of applications. In recent years, TMDs have been used as one of the building blocks in van der Waals heterostructures, leading to the development of efficient semiconductor components like highly scaled field-effect transistors,^1^ wide-range photodetectors,^2^ sensors,^3^ and flash memories.^4^ Although all mentioned types of devices are promising for next-generation electronics and optoelectronics, they need further development due to difficult-to-overcome processing steps, which affect the properties of thin TMD layers.

The critical problem in producing any 2D material-based devices is obtaining low-resistance contacts. One of the primary methods of controlling the current flowing through a metal/semiconductor junction is to align the metal work function to the semiconductor’s electron affinity to minimize the Schottky barrier height. In bulk semiconductors, low contact resistance is often facilitated by methods that can locally dope the area under the contact. This strategy is challenging to implement in the case of two-dimensional TMDs due to their atomic thickness. Moreover, widely used methods of producing contacts (e.g., resistive evaporation^5^ or e-beam evaporation)^6^ damage the structure of TMD materials and lead to the Fermi level pinning (FLP) at the metal/semiconductor junction. This effect makes Schottky barrier height, in fact, independent from metal work function.^7^

Several approaches have recently been investigated to address this issue, and the FLP effect has even been used to create new strategies for contact engineering in TMD-based devices. One of the most promising is weakening the FLP at the metal/TMDs interface by suppressing metal-induced gap states (MIGS) with semimetal contacts. For instance, bismuth has near-zero DOS at the Fermi level, which is also positioned close to the conduction band of tungsten disulfide (WS_2_) and molybdenum disulfide (MoS_2_), resulting in ultralow contact resistance at the Bi/WS_2_ and Bi/MoS_2_ junctions.^8^ Another example involves traditional substitutional doping techniques with Cl-dopants^9^ or chemical surface molecular n-dopants,^10^ which can passivate sulfur vacancies at the interface and thus move the position of the Fermi level. Few layer graphene^11^ and indium^12^ van der Waals contacts also exhibit a highly depinning nature due to a lack of interface states and covalent bonds to MoS_2_ and WS_2_. A similar strategy is applied by using buffer layers (interlayers), which can reduce contact resistance by preventing interaction between the metal and 2D semiconductor. For example, a dielectric layer of transition metal oxide TiO_2_ has been used to unpin the Fermi level in field-effect transistors based on WS_2_,^13^ reducing the contact resistance by over 18 times. Recently, even thin layered semiconductors (WSe_2_, MoSe_2_)^14^ and insulators (hBN)^15,16^ have been used as interlayers, creating van der Waals heterostructure-based devices with a lowered contact resistance from 55% even up to 90% in comparison to devices without interlayer. Usually, in reported studies, the interlayer is located both under the contact and in the FET’s channel. Without comparing the devices in which the interlayer is present only in the contact area and not in the channel, it is difficult to determine the actual impact of the interlayer on the current flowing through the metal/2D material junction. The lack of such studies results from challenges in the fabrication of complex devices (e.g., with heterostructures only in the contact area) caused by the necessity of using selective etching, which removes individual layers from specific areas of the heterostructure. However, selective etching of heterostructures with atomic precision, which can address each layer of the heterostructure individually, is still in development. Currently, selective etching methods for layered materials are mainly applied to graphene^17,18^ and hBN^19^ layers, there is a lack of studies performed on TMDs. There have been two alternative methods reported for selective etching. In one, layers of Nb-doped WSe_2_ with a thickness of 21 nm were patterned and etched into suitable shapes, then transferred using PDMS onto WSe_2_.^20^ In another, 40 nm thick wheel-shaped MoS2 layers were transferred onto the MoS2 monolayer using a silicon nitride membrane with a gold adhesion layer.^21^ Moreover, other studies show heterostructures based on TMDs monolayers with defined shapes with laterals sizes from 10 to 100 μm.^22,23^ However, to the best of our knowledge, no studies have been published where selective etching is replaced by a method that allows the fabrication of heterostructures composed of TMDs monolayers with arbitrary shapes down to 100 nm in lateral dimensions.

Moreover, those new transfer strategies and, thus, the reduction of contact resistance, are often evaluated based on the performances of only a few devices. This may hide issues related to the performance reproducibility and potential inhomogeneity of 2D materials. There is a lack of more extensive studies on this topic, with conclusions based on statistical measures of a large number of devices, which is particularly crucial when investigating complex 2D material-based structures.

In this work, we are the first to demonstrate a Au/MoS_2_/WS_2_ junction to effectively reduce contact resistance in monolayer WS_2_-based field-effect transistors. To enable this, we propose a new fabrication approach using a gold tape based transfer method, allowing the creation of numerous monolayer-based van der Waals heterostructures in one transfer process with lateral dimensions even down to 100 nm. In our devices fabricated with gold-assisted transfer, the MoS_2_ monolayer, acting as an under-contact interlayer (UCI), which is a layer directly between the channel (WS_2_) and metal contact (Au), enables using FLP to our advantage for achieving favorable band alignment at the junction. To access reproducibility and thoroughly investigate the influence of the UCI on the device’s performance, we examined key FET performance indicators of 160 devices (80 devices with and 80 devices without MoS_2_ UCI). In the designed devices with a UCI, the channel of the FET consists only of the WS_2_ monolayer, while the MoS_2_/WS_2_ van der Waals heterostructure is located only below the contact area of the device. This architecture of the FET allows for a reliable determination of the contact resistance, which decreased by over 60% due to the influence of the MoS_2_ UCI.

## Results and Discussion

**Figure 1:**
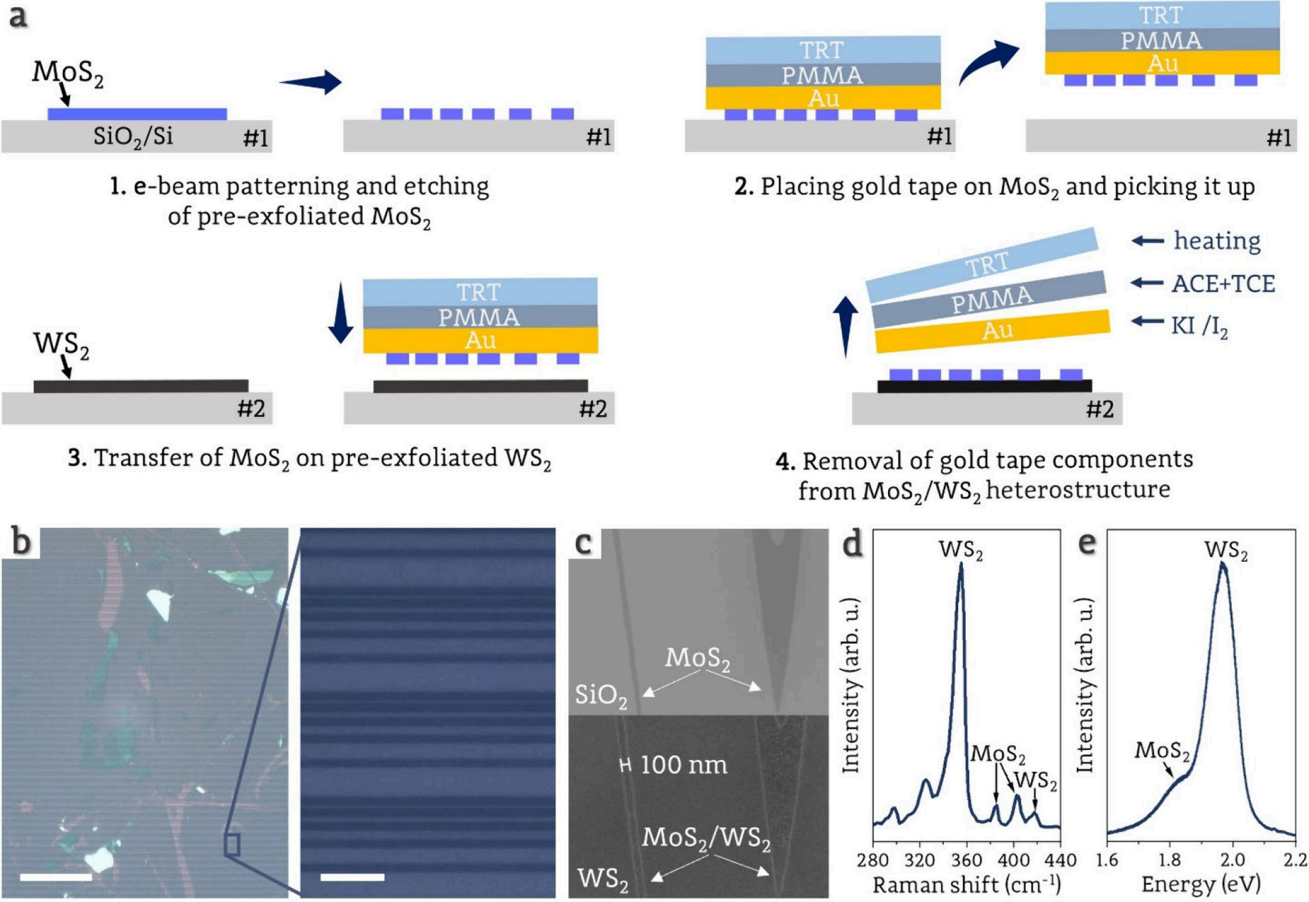
a) Simplified schematic representation of the gold-assisted transfer process. #*n* represents different SiO_2_/Si substrates (the detailed process is described in the Methods section). b) An example of heterostructure with monolayer MoS_2_ stripes transferred on continuous WS_2_ monolayer. The scale bar on the left picture is 150 μm, and on the right picture is 10 μm. c) SEM image of patterned and etched MoS_2_ monolayer on SiO_2_/Si substrate (Top image) and MoS_2_ monolayer transfer on WS_2_ monolayer on SiO_2_/Si substrate (Bottom image). The images show that the edges are well-preserved after the transfer and that a 100 nm-wide MoS_2_ stripe was successfully transferred. d) Raman spectrum and e) photoluminescence spectrum of MoS_2_/WS_2_ heterostructure with marked peaks corresponding to the individual component layers.

Devices with and without under-contact interlayer (UCI) were fabricated on SiO_2_/Si substrate using monolayers of MoS_2_ and WS_2_ prepared with gold-assisted mechanical exfoliation (as described in the “Methods” section). To create the unique heterostructures consisting of MoS_2_ stripes on the WS_2_ monolayer, we developed an approach using the gold-assisted transfer method (Figure 1a), which allows for the transfer of prepatterned monolayers with shapes defined by e-beam lithography and etching (details provided in the “Methods” section). The transfer method is possible because of the higher binding energy of MoS_2_ to Au than to SiO_2_ substrate.^24^ Moreover, the nobility of Au and interfacial strain between Au and MoS_2_^25^ facilitates the detachment of the MoS_2_ monolayer from SiO_2_. In our work, the MoS_2_ monolayer was patterned and etched to form stripes with varying spacings for contact resistance investigation using the transfer length method (TLM). MoS_2_ stripes were transferred with gold tape on a continuous WS_2_ monolayer. The optical image presented in Figure 1b illustrates the capability of gold-assisted transfer to fabricate numerous heterostructures during a single transfer (on the single substrate), covering a total area of structure exceeding 500 000 μm^2^. With our method, in which we are using layers shaped by the e-beam lithography technique, it is possible to create heterostructures with precisely defined edges and lateral sizes even down to 100 nm (Figure 1c), making it suitable for scale-down devices. The Raman and photoluminescence spectra of one of the heterostructures consisting of monolayer MoS_2_ and WS_2_ are shown in Figures 1d,e. More extensive analysis, including Raman mapping, AFM, and SEM images, showcasing the transfer of monolayers is presented in Supporting Information 1.

**Figure 2:**
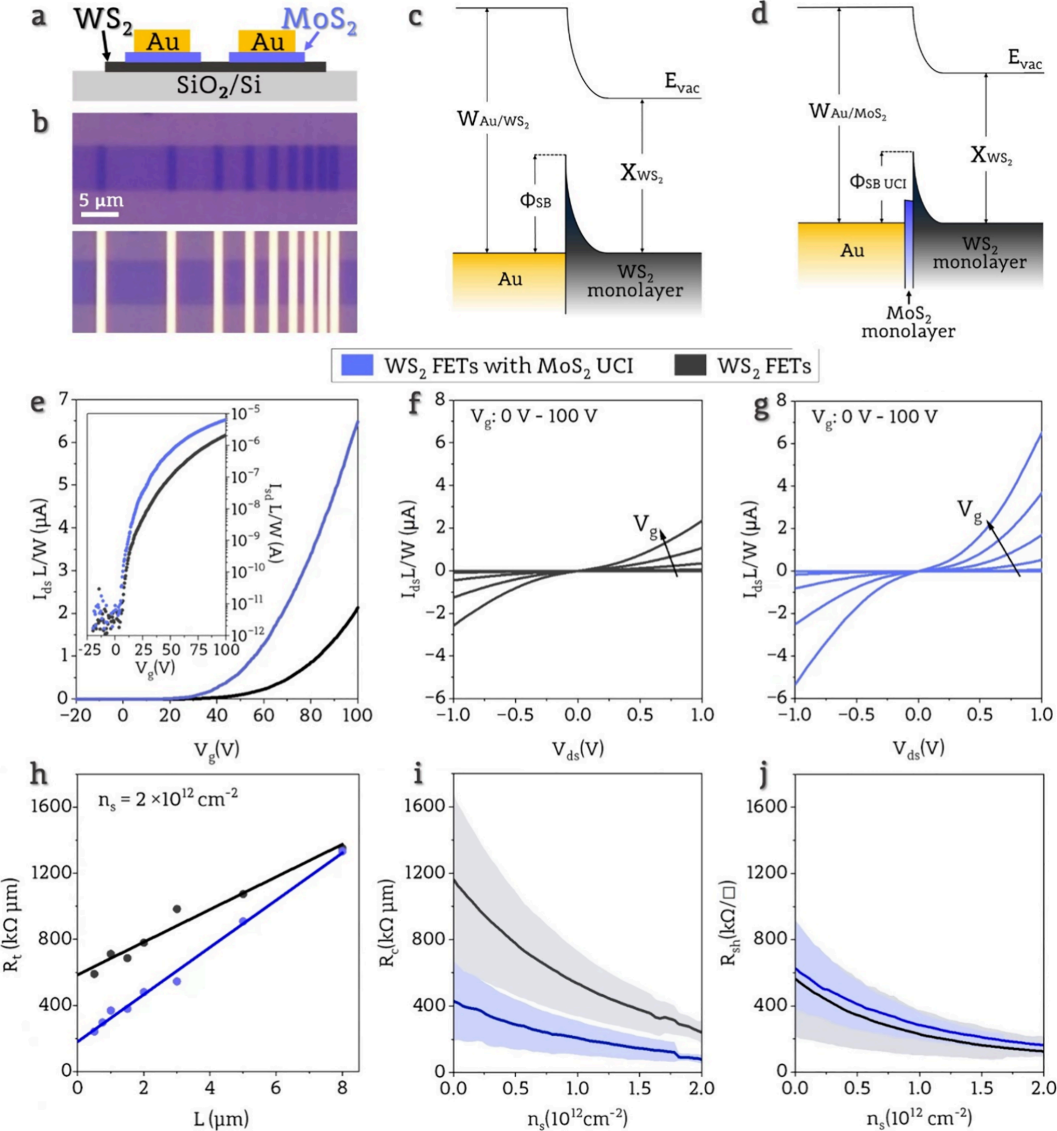
a) Schematic of the device with UCI. b) An optical image of the heterostructure with various spacing between MoS_2_ stripes placed on the WS_2_ layer (Top image). An optical image of the device shows gold contacts evaporated on the heterostructure area, creating a configuration that enables the use of the TLM method (Bottom image). c) Schematic representation of band alignment at Au/WS_2_ junction. d) Schematic representation of band alignment at Au/MoS_2_/WS_2_ junctions. e) Normalized transfer characteristics plotted on a linear scale, comparing representative devices with and without UCI. The inset shows the same results on a semilogarithmic scale. Measurements were performed with *V*_ds_ = 1 V. f) Normalized output characteristics for different gate voltages from 0 to 100 V, with a 20 V step for a representative device without a UCI, and g) for a representative device with a UCI. h) Total contact resistance *R*_t_ dependence on channel length *L* calculated for carrier concentration of *n*_s_ = 2 × 10^12^ cm^–2^ for two representative TLM sets with fitted linear function for *R*_c_ extraction. (i) Mean value and standard deviation of contact resistance (*R*_c_) as a function of carrier concentrations. j) Mean value and standard deviation of sheet resistance (*R*_sh_) as a function of carrier concentrations.

In order to fabricate field-effect transistors, Au contacts were thermally evaporated on MoS_2_/WS_2_ heterostructure areas, creating Au/MoS_2_/WS_2_ junctions (Figure 2a,b). In the designed devices with UCI, the MoS_2_ stripes have a width of 1.3 μm, while the gold contacts have a width of 1 μm to ensure no direct contact between Au and WS_2_. Channel lengths (*L*) are 0.5, 0.75, 1.5, 2, 3, 5, and 8 μm, which are strictly related to the arrangement of transferred MoS_2_ stripes. The channel width (W) is 6 μm and remains the same in all devices. To see the influence of the UCI on the performance of devices, we also prepared devices only with the WS_2_ monolayer (without the MoS_2_ as a UCI) with the same dimensions of the channel and contacts. Considering that both types of devices were not prepared on exactly the same WS_2_ monolayer, it was necessary to exclude differences in the fabrication processes. In the case of devices with UCI, gold was used to both exfoliate and transfer, so it was important to ensure that the gold was removed entirely after fabrication. If the etching of the gold layer is performed poorly, then it can leave behind residues that dope the TMDs monolayer,^26^ leading to a change in FET properties. To prove the elimination of the effects related to the fabrication, we performed XPS analysis (see Supporting Information 2), which revealed that the presence of the Au 4f doublet line in the spectra was below the detection limit estimated for 0.01% of atom concentration in the surface region. This demonstrates that the cleaning process is effective, and any expected differences in the performance of devices with and without UCI should not be attributed to gold residues.

Fabricated devices were electrically characterized in a back-gate configuration under high vacuum conditions. Transfer characteristics for representative devices with and without a UCI are shown in Figure 2e. Characteristics were normalized by the channel length and width. Devices with a UCI exhibit a lower threshold voltage and higher on-state current, which indicates that the MoS_2_ UCI enhances the performance of the devices. In Figure 2f,g, output characteristics demonstrate source-drain current three times higher for devices with UCI. Meanwhile, the shape of characteristics of both types of devices remains nonlinear and corresponds to the typical current–voltage dependence in FETs with Schottky contacts. It is commonly observed that the Fermi level pinning is present at the metal/2D-TMD junctions, affecting the Schottky barrier height at the interface. The level of pinning in a system consisting of specific TMD and various metals is almost independent of the metal’s work function,^27^ which makes FLP a crucial issue in designing 2D FETs. However, when junctions with a specific metal and various TMDs are compared, the work function of the combined system (metal and TMD layer) changes significantly with different TMDs. Consequently, the pinning level also varies considerably depending on the used TMD.^28^ In such a case, the Schottky barrier height (ϕ_SB_) at the junction will depend on the work function of the combined system (level of pinning, *E*_FP_) and the electron affinity of the TMD layer (*Χ*),^14^ as in eq 1.
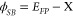
1

Previously in the literature, it was confirmed that the FLP effect is occurring at the Au/MoS_2_ and Au/WS_2_ interfaces, but the level of pinning is preset at different energy levels in those junctions.^29,30^ In Figure 2c,d, we schematically show band diagrams of Au/MoS_2_/WS_2_ and Au/WS_2_. The work function of the combined system of Au/MoS_2_ has a value lower than the work function of the Au/WS_2_ system. Moreover, the FLP effect is layer-number dependent and strongly affects only the first layer of the material in contact with the metal.^31^ Therefore, we expect that by using the MoS_2_ interlayer and creating the Au/MoS_2_/WS_2_ junction, we are mitigating FLP and achieving lower Schottky barrier height, which leads to reduced *R*_c_. Furthermore, we are anticipating that the mismatch between the conduction bands of MoS_2_ and WS_2_ is creating a step-like offset at the interface of those materials (Figure 2d), which can reduce contact resistance and improve the efficiency of the mechanism responsible for the thermionic transport occurring through the Au/MoS_2_/WS_2_ junction.

To verify our predictions, we investigated 80 devices with UCI and 80 devices without UCI. We calculated the total resistance of devices based on data from the linear operation regime of transfer characteristics of 13 TLM structures of each type and used it to extract contact resistance and sheet resistance (from channel resistance, *R*_ch_) based on eq 2 and 3.
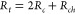
2
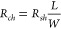
3

Representative results of total resistance *R*_t_ calculated for the gate voltage corresponding to the carrier concentration of *n*_s_ = 2 × 10^12^ cm^–2^_,_ at which devices are in on-state (linear operation regime), are presented in Figure 2h for representative TLM sets of both types of devices (formulas and detailed graphs are demonstrated in Supporting Information 3). Figure 2i,j shows extracted mean values and standard deviations of *R*_c_ and *R*_ch_ for devices with and without UCI for different carrier concentrations *n*_s_. *R*_c_ and *R*_ch_ strongly depend on *n*_s_. Carrier concentration in the FET’s channel is modulated by gate voltage, which also affects the interface between channel material and contact. This effect, known as contact-gating, occurs in devices with global back-gate geometry and lowers the Schottky barrier width at the junction by applied back-gate voltage, thus allowing carrier tunneling through the barrier and reducing *R*_c_.^32^ For all values of carrier concentration, the contact resistance of the Au/MoS_2_/WS_2_ junction is around 60% lower than the contact resistance of the Au/WS_2_ junction. This supports our prediction that using MoS_2_ UCI can create a more favorable band alignment, reducing contact resistance. Comparing the values for *n*_s_ = 2 × 10^12^ cm^–2^, at which devices are in on-state, we achieved mean *R*_c_ values of 242 kΩ·μm with a standard deviation of 72 kΩ·μm for devices without UCI, which are comparable to other studies^33,34^ and 79 kΩ·μm with a standard deviation of 27 kΩ·μm for devices with a UCI. A similar reduction of *R*_c_, but based on a different Schottky barrier-lowering mechanism, was observed in WS_2_ devices with graphene interlayer and Ni contacts^34^ (more extended comparison is presented in Supporting Information 4). However, in the case of our devices with UCI, we are not unpinning the Fermi level, but by using the MoS_2_ monolayer, we create a junction with a more advantageous energy landscape using the FLP effect in our favor. Moreover, to ensure that the decrease in *R*_c_ is not attributed to the extension of contact length and simultaneous reduction of channel length, we considered the possible impact of an additional 150 nm long MoS_2_ monolayer extending beyond each side of the contact on *R*_c_. We performed the analysis presented in Supporting Information 5, which confirms that the observed reduction cannot be attributed to changes in the effective geometry of the device.

**Figure 3:**
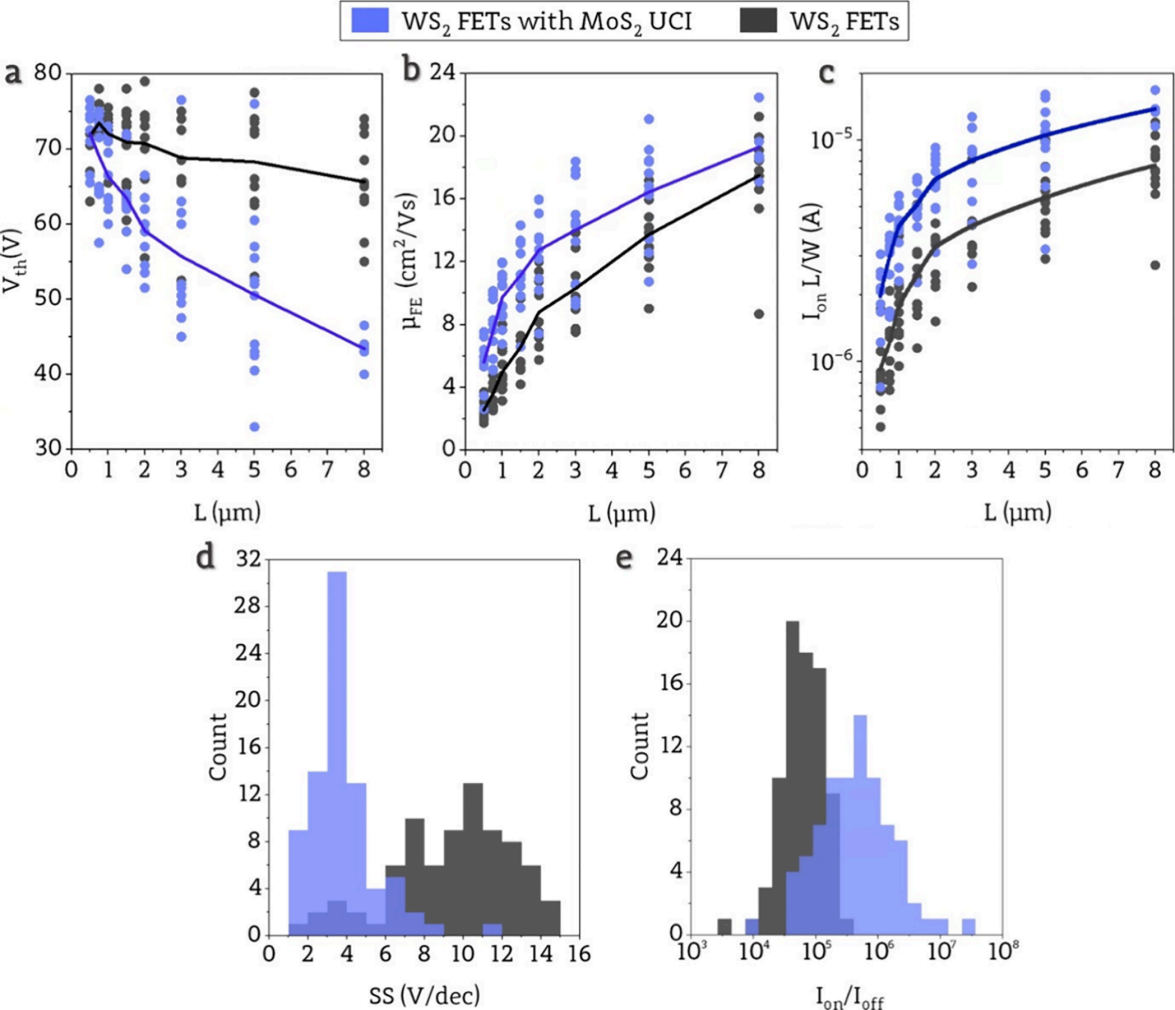
Distribution of a) threshold voltage, b) field-effect carrier mobility, and c) normalized on-state current (*I*_on_) for different channel lengths for devices with and without UCI. Lines represent mean values. d) Histograms showing the variation in subthreshold swing (SS) and e) on-state current to off-state current ratio for devices with and without UCI.

To investigate device-to-device variation in a more statistical approach and assess the impact of contact resistance reduction on FETs performance, in Figure 3, we presented crucial parameters such as threshold voltage (*V*_th_), field-effect mobility (μ_FE_), subthreshold swing (SS), and on- to off-state current ratio (*I*_on_/*I*_off_) (details in Supporting Information 6). Figure 3a shows the distribution of *V*_th_ as a function of channel length (*L*) and corresponding mean values connected with lines as guides to the eye. High *V*_th_ values result from 285 nm thick SiO_2_. For devices without a UCI, values of *V*_th_ are close to 70 V with a slight dependence of *L* compared to *V*_th_ of devices with a UCI, which exhibits a significant dependence of *L*. The negative shift and change in dependency on channel length can be attributed to different Schottky barrier heights in Au/WS_2_ and Au/MoS_2_/WS_2_ junctions. A high Schottky barrier leads to an increased *V*_th_, indicating that the barrier at the Au/WS_2_ junction is anticipated to be higher than that at the Au/MoS_2_/WS_2_ junction. The dependence of *V*_th_ on the channel length can be related to the significant *R*_c_ value. When the channel length is scaled down, the influence of *R*_c_ becomes more substantial, ultimately becoming the primary factor restricting device performance as it dominates the total device resistance,^35^ resulting in higher *V*_th_ for shorter channels.

A similar influence of *R*_c_ is seen in field-effect mobility (μ_FE_) values (Figure 3b), which show strong channel length dependence, commonly observed due to the effect of Fermi level pinning existing in TMDs/metal junctions.^36^ Field-effect mobility values are higher for devices with a UCI for each channel length, which may initially seem unusual, because the channel area is the same in both types of devices and consists only of the WS_2_ monolayer. In fact, μ_FE_ depends not only on the intrinsic properties of WS_2_ but also on the properties of the entire device, which is why a strong influence of the contact resistance can be noticed. Higher values of μ_FE_ indicate reduced contact resistance in devices with UCI. This observation is supported by *I*_on_ values (Figure 3c) that are greater for devices with UCI and strongly dependent on *L* despite being normalized by channel dimensions. For both types of devices, μ_FE_ reaches almost 20 cm^2^/(V s), which is comparable with other studies performed on WS_2_-based devices.^36,37^Figure 3d shows the distribution of SS. The large SS values for both types of devices result from using a 285 nm thick gate oxide layer. Devices without UCI exhibit SS values with the mode at 11 V/dec, whereas for devices with UCI, the SS is over 3 times lower with the mode value of 3 V/dec. The SS parameter is calculated across voltages lower than the threshold voltage and represents the rate at which the device switches from the off-state to the on-state. Hence, when a higher Schottky barrier and consequently increased contact resistance are encountered, a higher SS can be expected, hindering the flow of charges through the junction. Figure 3e shows the distribution of *I*_on_/*I*_off_ with a mode value for devices with UCI at the level of 10^6^ and for devices without UCI at 10^5^. This supports the previous observation that current in devices without UCI is limited. SS and *I*_on_/*I*_off_ ratios show no dependency on channel length for both types of devices with and without UCI (Supporting Information 7).

**Figure 4:**
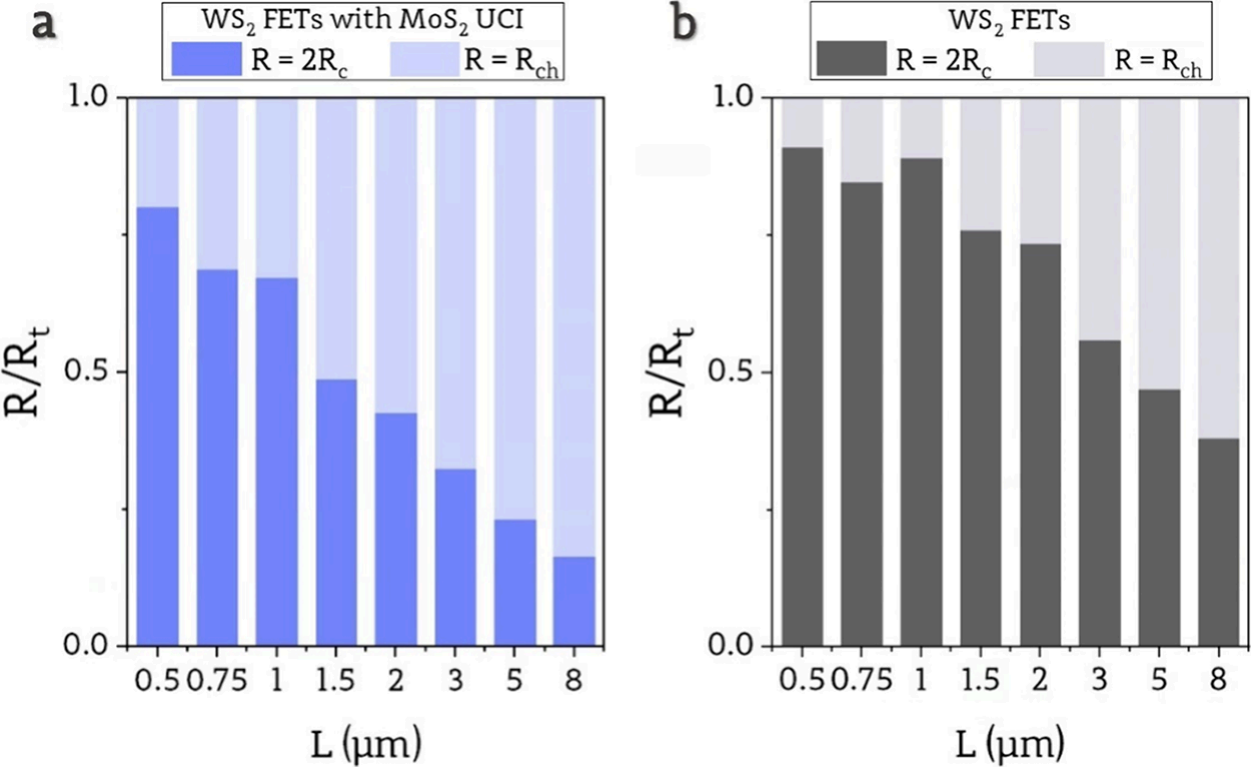
a) Stacked bar plots of *R*_ch_ and *R*_c_ contribution to the total resistance of devices with UCI and b) without UCI.

To further investigate the influence of contact resistance on devices’ performance, we illustrate the contribution of contact resistance and channel resistance (*R*_ch_), calculated with mean values using eq 3, to the mean total resistance of the device in Figure 4a,b for different channel lengths.

*R*_c_ and *R*_ch_ are independent of the channel length. Simultaneously, *R*_ch_ is proportional to channel length, which leads to varying impacts of 2*R*_c_ on *R*_t_ for long and short channels. For both types of devices, the contribution of 2*R*_c_ to *R*_t_ is much more significant for shorter channels. Furthermore, 2*R*_c_/*R*_t_ shows a stronger channel length dependence in devices with UCI. In contrast, devices without UCI, even with the longest channels, exhibit almost half of the total device resistance being caused by effects occurring on Au/WS_2_ junctions. This observation aligns with the analysis of *V*_th_ and μ_FE_ in Figure 3a,b, which demonstrated that these parameters vary with different channel lengths, which is directly related to the impact of contact resistance on the operation of devices. Additionally, the channel length dependence for those parameters was weaker in devices without UCI, consistent with the result in Figure 4, which indicates that 2*R*_c_’s contribution to *R*_t_ remains substantial across all channel lengths, while for devices with UCI, it drops significantly due to reduced *R*_c_.

## Conclusions

In this study, we are the first to report the enhancement of the performance of monolayer WS_2_-based FETs by using a monolayer of MoS_2_, which serves as an under-contact interlayer. To create MoS_2_/WS_2_ van der Waals heterostructure, we developed a gold-assisted method that enables the transfer of prepatterned TMD monolayers with lateral dimensions even down to 100 nm. With our method, we can fabricate numerous heterostructures with a total area exceeding 500 000 μm^2^ in one transfer process. Our approach enables us to reduce contact resistance by over 60% due to favorable band alignment at the Au/MoS_2_/WS_2_ junction. Statistical analysis of 160 FETs with and without an UCI highlighted the influence of contact resistance on the operation of devices both in the contact area and in the channel. Therefore, the further development of new strategies is needed to reduce this significant factor in order to achieve high-performance TMD-based electronics and optoelectronics.

## Methods

### TMD Monolayer Preparation

Large-area monolayers of MoS_2_ and WS_2_ were fabricated using gold-assisted exfoliation.^38^ Si wafers (Cemat Silicon) were cleaned by using argon plasma treatment (Diener Zepto). The plasma process was held for 10 min with 5 sccm gas flow and power of 4 W. Cleaned Si wafers were vacuum annealed at 300 °C for 3 h in a Kurt J. Lesker Nano 36 chamber followed by thermal evaporation of 100 nm gold layer. A 1 μm thick PMMA protective layer was spin-coated on the gold surface and baked for 2 min at 150 °C before the wafers were cleaved into smaller pieces. Thermal release tape (TRT Revalpha 3195MS) was placed on the PMMA layer. The bulk of chosen TMDs (2D Semiconductors) was prepared by peeling off the oxidized top layer of the crystal using Nitto tape. Gold tape (TRT/PMMA/Au) released from the Si substrate was immediately applied to the surface of the bulk crystal and gently peeled off. The gold tape with an exfoliated layer was placed on a 285 nm SiO_2_/Si substrate (Process Specialties). Samples were annealed at 150 °C for 10 min to peel off TRT and ensure strong adhesion of the exfoliated layers to the substrate. To remove PMMA and TRT residues, samples were soaked in trichloroethylene (TCE) at 50 °C for 10 min, then in acetone at 50 °C for 10 min, and rinsed in isopropanol. To remove the gold layer, samples were soaked in standard gold etchant (Sigma-Aldrich) for 5 min at room temperature and rinsed in two separate isopropanol solutions (10% IPA in DI water) and isopropanol. To remove left organic residues, WS_2_ samples were vacuum annealed at 350 °C for 3 h. This step was omitted in MoS_2_ samples to retain weaker adhesion to the substrate.

### Fabrication of van der Waals Heterostructures

Samples with the MoS_2_ monolayer were patterned with e-beam lithography (Raith e-Line Plus) and etched with an O_2_ RIE plasma (Oxford Plasmalab 80 Plus). After the removal of the resist layer used for the lithography process, gold tape (fabrication described in the previous section) is placed on the sample, covered with a glass slide, and loaded with a weight of 120 g for 20 min to achieve strong adhesion between the gold layer and patterned MoS_2_. In the next step, the weight is removed, and the sample with gold tape on top is placed on a hot-plate and annealed at 150 °C for 20 min. TRT peels off under the temperature, and the PMMA/Au stack stays on the substrate. After the annealing, when a sample cools down, another TRT is placed on the sample. A patterned MoS_2_/Au/PMMA/TRT stack is peeled off using tweezers. The stack is placed on a monolayer WS_2_ sample, then covered with a glass slide, loaded with a weight of 120 g, and left for 20 min. Next, the weight is removed, and the sample with the stack is placed on a hot-plate and annealed at 150 °C for 20 min. The TRT peels off due to temperature, and other components of gold tape are removed the same way as after exfoliation, as described in the previous section.

### Field-Effect Transistors Fabrication

Devices were fabricated in the back-gate configuration on a SiO_2_/Si substrate (285 nm thick silicon oxide) using e-beam lithography (Raith e-Line Plus) and O_2_ RIE etching (Oxford Plasmalab 80 Plus). In the case of devices with UCI, contacts were patterned only on the MoS_2_/WS_2_ heterostructure area. 70 nm pure Au contacts were thermally evaporated using a Kurt J. Lesker Nano 36, followed by a lift-off process.

### Electrical Characterization

Electrical characterization was performed using a DL Instruments 1211 Current Preamplifier, National Instruments DAQ 6366, and source measuring unit Keithley 2450. Measurements were carried out in a vacuum (1e-6 mbar) in an Oxford MicrostatHe2 cryostat at room temperature. We performed electrical characterization using the standard two-probe method, applying voltage and sensing current. Before the measurements, the samples were kept in a vacuum for 19 h, during which they were thermally annealed for 13 h at 200 °C.

### Raman Spectroscopy and Photoluminescence Measurements

Raman spectroscopy and photoluminescence measurements were carried out using a Renishaw inVia Qontor Raman spectrometer with a 532 nm excitation wavelength. Spectra were measured using a 100× objective with 1800 lines/mm grating. Laser power was kept below 0.5 mW to avoid damaging the material.

### XPS

The XPS system was equipped with a hemispherical energy analyzer EA 125 (Omicron), an RS40B1 (Prevac) source, and an RMC50 monochromator (Prevac); monochromatic radiation of 1486.6 eV (Al Kα) was used. The peak fitting procedure was supported by Casa XPS software.

## Supporting Information Available

SEM, AFM, and Raman characterization of MoS_2_/WS_2_ van der Waals heterostructure fabricated with gold-assisted transfer. Moreover, we include an evaluation of the removal of the gold layer after transfer based on XPS analysis. We also present a formula for extracting key FET parameters, a showcase of complete results of *R*_ch_ and *R*_c_ for all devices, and SS and *I*_on_/*I*_off_ ratio dependence on channel length (PDF)

## Abbreviations

TMDs: Transition metal dichalcogenides
FET: Field-effect transistor
MoS_2_: Molybdenum disulfide
WS_2_: Tungsten disulfide
FLP: Fermi level pinning
XPS: X-ray photoelectron spectroscopy
TRT: Thermal release tape
TCE: Trichloroethylene
