# Supplementary material for: Contact Resistance Engineering in WS2-Based FET with MoS2 Under-Contact Interlayer: A Statistical Approach
Source: ACS Appl Mater Interfaces. 2024 Aug 26;16(36):48556–64. doi: 10.1021/acsami.4c09688 (PMC11403553; doi:10.1021/acsami.4c09688)
Supplement: Supplementary file 1 — am4c09688_si_001.pdf [file am4c09688_si_001.pdf]

Supporting Information to:

Contact resistance engineering in WS_2-based FET with MoS_2 under-contact interlayer – a statistical approach

Małgorzata Giza[1], Michał Świniarski[1], Arkadiusz P. Gertych[1], Karolina Czerniak-Łosiewicz[1], Maciej Rogala[2], Paweł J. Kowalczyk[2], Mariusz Zdrojek[1]***

[1] Faculty of Physics, Warsaw University of Technology, Koszykowa 75, 00-662 Warsaw, Poland

[2] Faculty of Physics and Applied Informatics, University of Łódź, Pomorska 149/153, 90-236 Łódź, Poland

*malgorzata.giza.dokt@pw.edu.pl

**mariusz.zdrojek@pw.edu.pl

Supporting Information 1: Characterization of van der Waals heterostructures fabricated with gold-assisted transfer

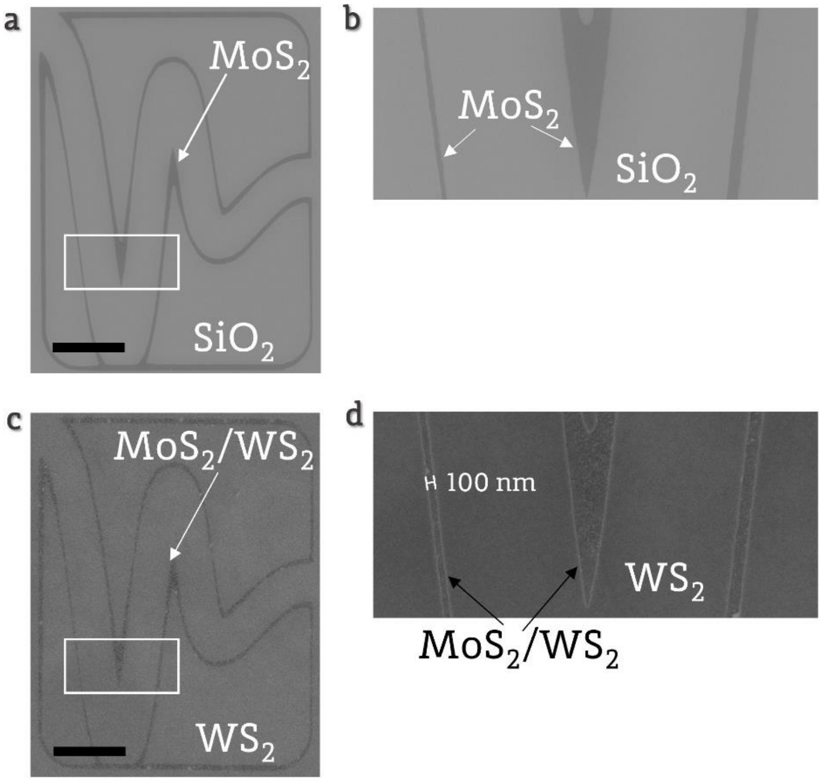

Figure S1 a) SEM images of pattern and etched MoS_2 monolayer on SiO_2/Si substrate representing the Faculty of Physics WUT logo. The black scale bar is 4 µm. b) Zoom-in picture of the area outlined by the white rectangle in Figure S1a. c) SEM images of logo transfer with gold-assisted method on WS_2 monolayer creating MoS_2/WS_2 van der Waals heterostructure. The black scale bar is 4 µm. d) Zoom-in picture of the area outlined by a white rectangle in Figure S1c. The picture shows that it is possible to transfer a monolayer of lateral size down to 100 nm using gold-assisted transfer. SEM images were taken using an InLens secondary electron detector in the Raith e-Line Plus system.

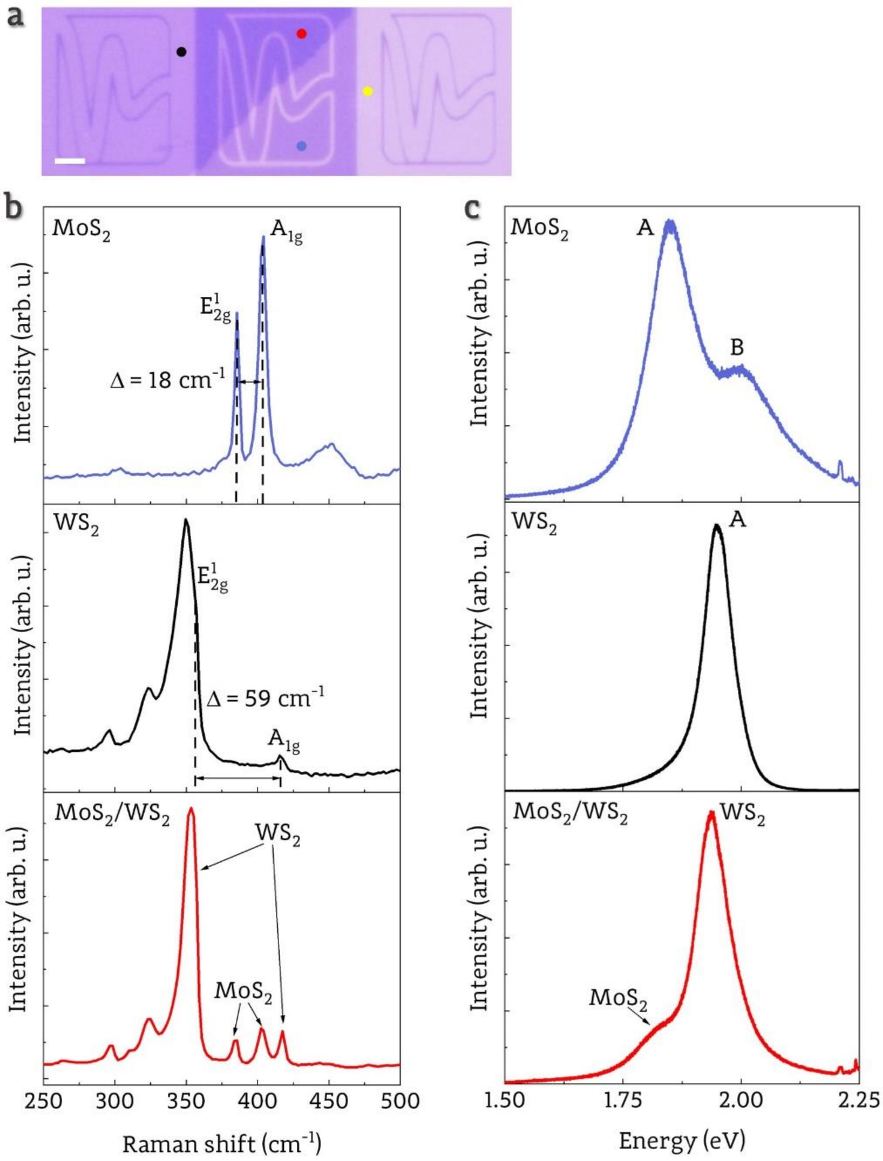

Figure S2 Raman and photoluminescence measurements of MoS_2/WS_2 heterostructure and component monolayers. a) Optical image of heterostructure representing Faculty of Physics WUT logo. The blue dot marks the area with only a MoS_2 monolayer, the black dot marks an area with only a WS_2 monolayer, and the red dot marks the area with MoS_2/WS_2 heterostructure. The yellow dot marks an area with a bare SiO_2/Si substrate. The scale bar is 4 µm. b) Raman spectra measured in the areas marked by dots in Figure S2a. The positions of the E^1_{2g} and A_{1g} modes in the MoS_2 and WS_2 spectra are marked with dashed lines and were determined by fitting Lorentzian functions to the data. The difference in peak positions was 18 cm^{-1} and 59 cm^{-1}, respectively, for the MoS_2 and WS_2 spectra, confirming that these are monolayers[1,2]. In the MoS_2/WS_2

heterostructure spectrum, the peaks corresponding to the component monolayers are marked with arrows. c) Photoluminescence spectra measured in the areas marked by dots in Figure S2a. MoS_2 spectrum exhibits A and B exciton peaks, and the WS_2 spectrum only exhibits A exciton peak, which is typical for the monolayer form of those materials[3,4]. In the MoS_2/WS_2 heterostructure photoluminescence spectrum, the peaks corresponding to the component monolayers are marked with arrows. All spectra were acquired with a 532 nm laser.

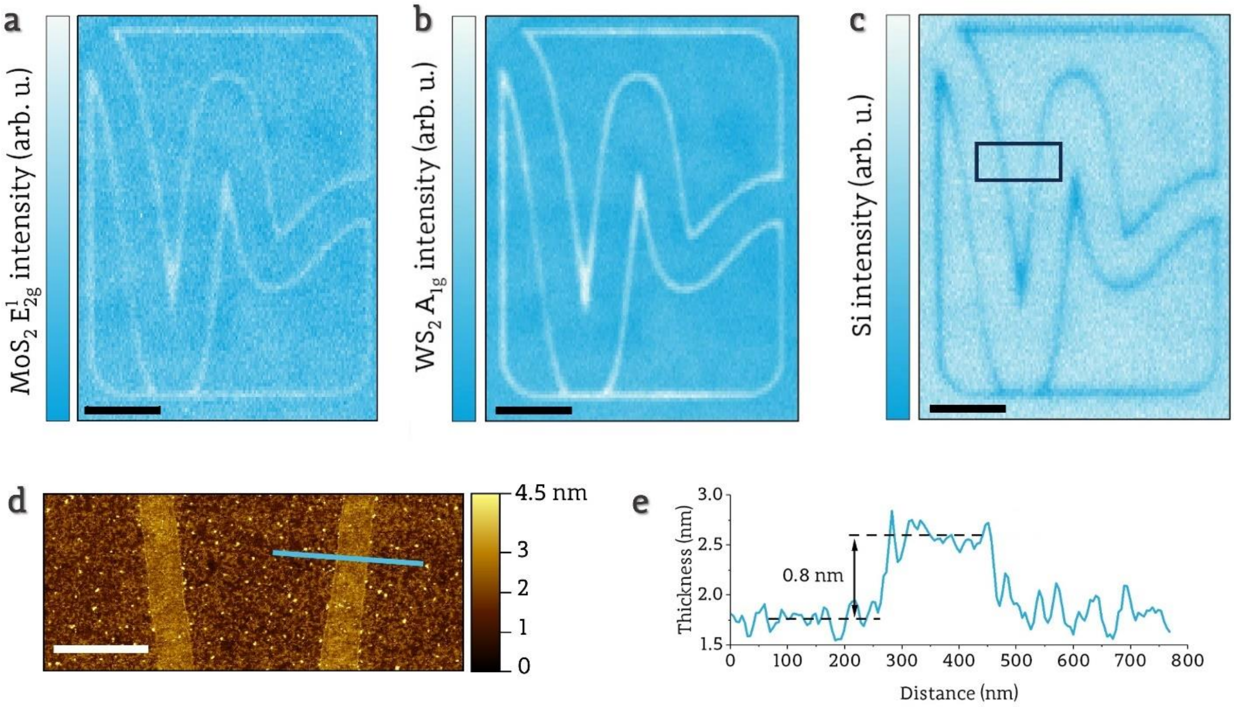

Figure S3 Raman mapping and AFM analysis of MoS_2/WS_2 heterostructure representing Faculty of Physics WUT logo. Maps represent Raman intensity signal for a) MoS_2 E^1_{2g} mode (382 cm^{-1}), b) WS_2 A_{1g} mode (417 cm^{-1}), c) Silicon mode (521 cm^{-1}). All scale bars are 4 µm. The reduced intensity of the Silicon mode in the heterostructure area corresponds with the stronger light absorption of the heterostructure. Raman MoS_2 intensity shows the appearance of monolayer MoS_2 on the sample. WS_2 intensity shows the appearance of monolayer WS_2 on the sample with increased intensity of A_{1g} mode in the heterostructure area due to interactions between MoS_2 and WS_2 layers[5]. d) AFM height image of heterostructure area

outlined by a black rectangle in Figure S3c. The white scale bar is 500 nm. e) Height profile across the blue line marked in Figure S3d. AFM scan was measured using Bruker Dimension Icon AFM in tapping mode.

Supporting Information 2. XPS analysis of MoS_2 and WS_2 monolayers fabricated with gold-assisted exfoliation

The XPS analysis was performed on four samples: a monolayer of MoS_2 prepared without an annealing step, a monolayer of MoS_2 prepared with an annealing step, and, similarly, on two samples with WS_2 monolayer. For all samples, the SiO_2/Si substrate was used. The presence of the Au 4f doublet line in the measured spectra was below the detection limit estimated in this case for 0.01% of atom concentration in the surface region. The estimation was carried out using the Si 2p line (close to 103 eV) and noise measured at 86 eV, where gold 4f peaks should occur. The Au 4f 7/2 and Au 4f 5/2 were incorporated (see insets in Figure S4), so that their intensity exceeded the level of the measured background. The surface area of these hypothetical peaks is more than 3000 times smaller than the surface area of the visible Si 2p line (for all investigated samples). Including in the calculation the relative sensitivity factor value for Au:Si, which is 17.1:0.8, and the fact that the measured atomic concentration of silicon in the surface layer was approx. 30%, which gives us the mentioned 0.01% concentration of maximal hypothetical gold atoms.

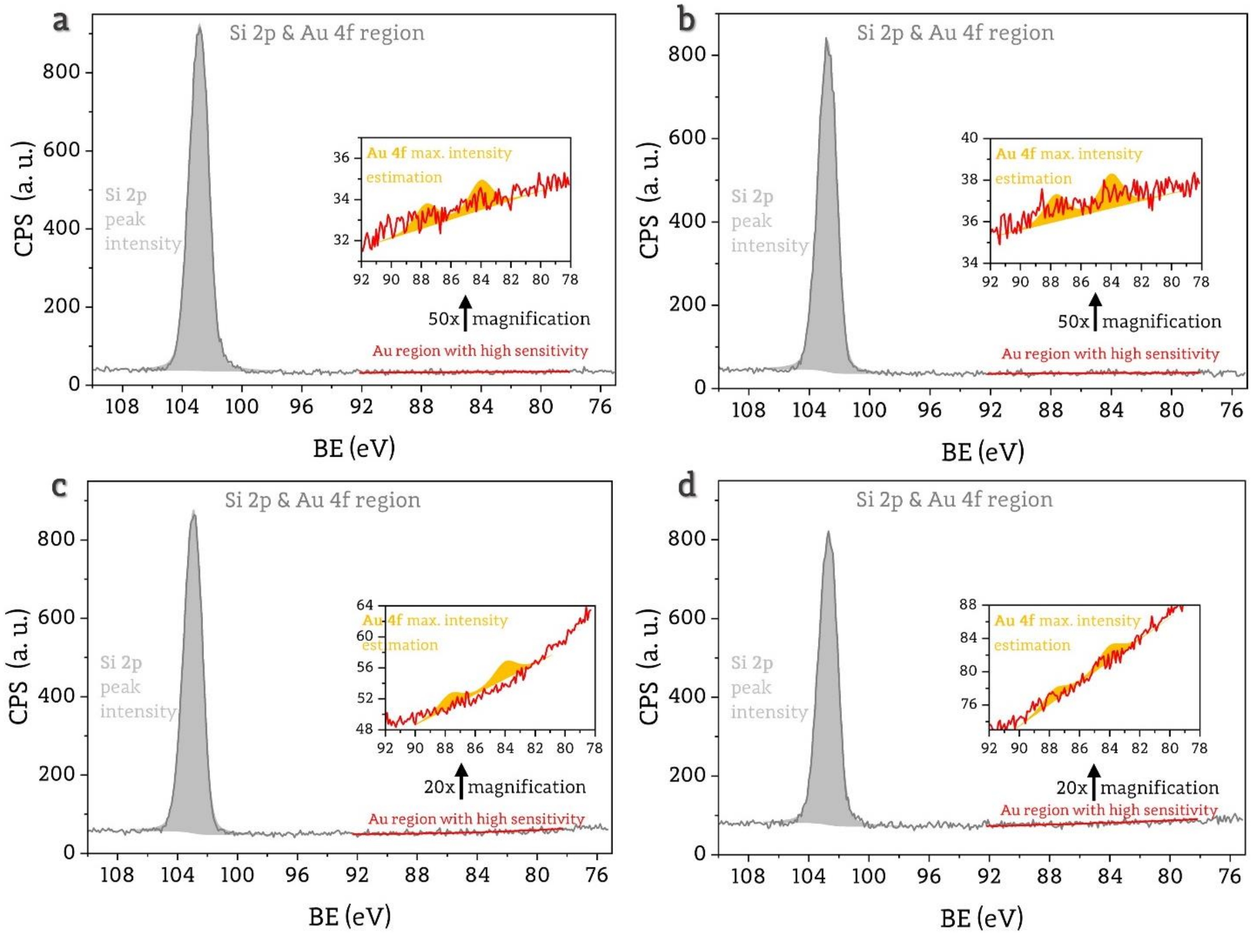

Figure S4 The XPS spectra of a) the MoS_2 sample prepared without annealing, b) the MoS_2 sample prepared with annealing, c) the WS_2 sample prepared without annealing, and d) the WS_2 sample prepared with annealing. All measurements were performed on the region covering Si 2p and Au 4f core lines (grey curve). The high sensitivity spectra focusing on the Au 4f line (with low noise) are additionally included (red curve). The insets present the magnification of intensity (CPS) near the Au 4f region. Au 4f peaks are not visible in the presented data. The maximal possible hypothetical Au peaks, however, were incorporated into the spectra to estimate the concentration of Au that could be hidden in the noise.

Supporting Information 3. Extraction of contact resistance and sheet resistance using the TLM method

On Figure S5a we present R_t distribution for each of 160 devices calculated for carrier concentration equal to 2×10^{12} cm^{-2} with Rt mean values. In order to extract Rc for both type of devices we follow specific steps listed below:

1. Determine the total resistance of each device in a single TLM set for various carrier concentrations.
2. Fit equation S1 to the data for a single TLM set for each carrier concentration separately.
3. Extract R_c from the intercept of the fitted equation.
4. Repeat steps 1-3 for each TLM set.
5. Calculate the mean value and standard deviation of Rc for each carrier concentration.

The showcase of all R_c and R_{sh} values extracted from measurements of 13 TLM structures with MoS_2 UCI and 13 TLM structures without UCI is presented in Figures S5b and S5c. Values were calculated based on the linear fitting of equation S1 to total resistance as a function of various channel lengths for each TLM set separately:

$$R_t(L) = 2R_c + R_{sh}\frac{L}{W} \quad \text{(S 1)}$$

R_t was calculated using V_{ds} of 1V (at which transfer characteristics were measured) divided by I_{ds} data from transfer characteristics at linear operation regime for different overdrive voltages (V_g-V_{th}).

Carrier concentration n_s was calculated from overdrive voltage:

$$n_s = \frac{C_{ox}(V_g - V_{th})}{q} \qquad \text{(S 2)}$$

C_{ox} is a gate oxide capacitance calculated from the equation S3:

$$C_{ox} = \frac{\varepsilon_0 \varepsilon_r}{d_{SiO_2}} \qquad \text{(S 3)}$$

ε_r is SiO_2 relative permittivity (3.9) and d_{SiO2} is SiO_2 thickness (285 nm).

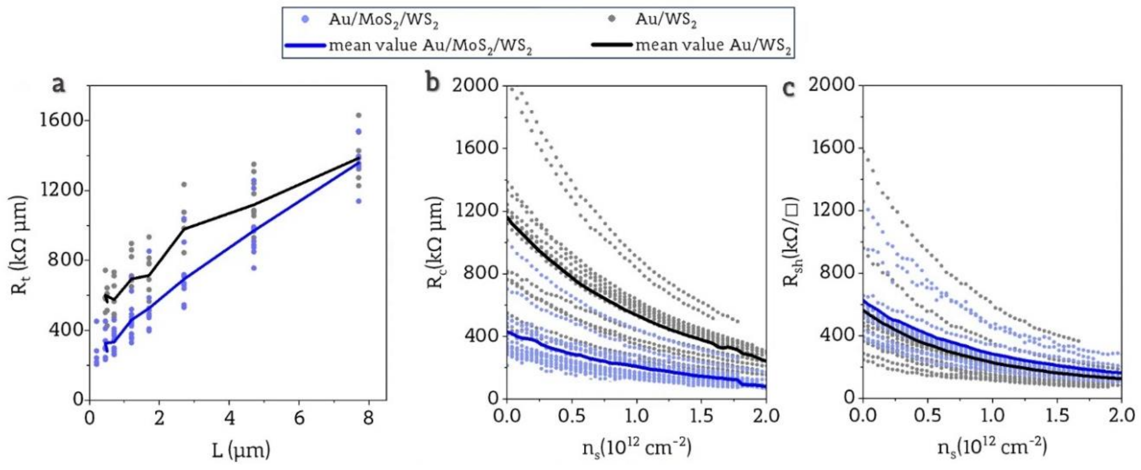

Figure S5 a) R_t distribution of 160 devices calculated for $n_s = 2\times10^{12}$ cm^{-2} with mean values connected by lines. b) R_c values and c) R_{sh} as a function of carrier concentrations for all measured TLM structures.

Supporting Information 4. Comparison with other studies

**Materials**	**WS_2 thickness**	**R_c (kΩ µm)**	**Ref. no**
$Au/MoS_2/WS_2$	monolayer	117	This work
Au/WS_2	monolayer	270	This work
$Ni/Graphene/WS_2$	14 nm	100	6
Ni/WS_2	14 nm	350	6
$Ti/TiO_2/WS_2$	15 nm	24	7
Graphene 1D contact/WS_2	monolayer	20	8
$Au/In/WS_2$	-	2.4	9
$Au/Cr/WS_2$	5.2 nm	62.6	10
$Au/Ni/WS_2$	monolayer	2.1	11

Supporting Information 5. Impact of the effective geometry of devices with under-contact interlayer on contact resistance

To ensure that the decrease in contact resistance isn't caused by a reduction of the effective channel length of the devices due to the presence of 300 nm of additional MoS_2 monolayer in the channel (average 150 nm on each side - Figure S6a), we performed an analysis in which we assume that the channel lengths are shortened by 300 nm in every device with under-contact interlayer (Figure S6b). When we compare the R_c decrease calculated for shortened channels to the values calculated for devices in which we assume the MoS_2 monolayer, which extends beyond gold, is a part of the channel, we can see that reduction in R_c changes only by 7%. This suggests that a significant impact in the R_c reduction is caused not by shortened effective channel length but by the influence of the presence of the MoS2 interlayer on the junction in our field-effect transistors.

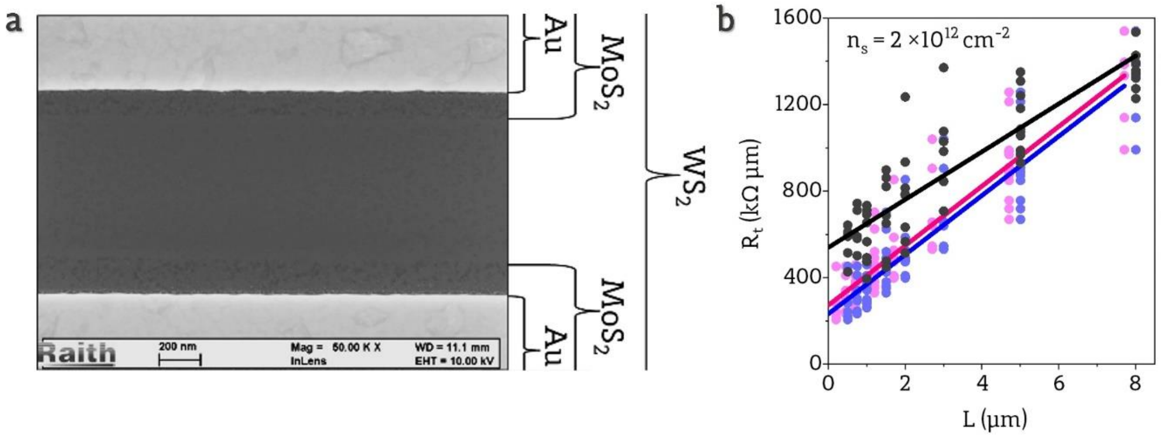

Figure S6 a) SEM image of the representative device with under-contact interlayer, which extends beyond gold contacts. b) R_t values and fitted linear functions for devices with an under-contact interlayer (blue), devices without an under-contact interlayer (black), and devices with an under-contract interlayer with the assumption that the channel lengths are shortened by 300 nm due to the presence of MoS_2 layer.

Supporting Information 6. Extraction of electrical parameters

Threshold voltage: We extracted V_{th} from transfer characteristics using the linear extrapolation method.

Field-effect mobility: μ_{FE} was calculated from the equation:

$$\mu_{FE} = \frac{dI_{ds}}{dV_g}\left(\frac{L}{WV_{ds}C_{ox}}\right) \qquad \text{(S 4)}$$

Subthreshold swing: SS was extracted from the slope of transfer characteristics in a logarithmic scale for one order of I_{ds} magnitude.

I_{on}/I_{off} ratio: I_{on} was extracted as a maximum I_{ds} value from transfer characteristics. I_{off} was calculated as the mean value of I_{ds} data for V_g ranging from -20 V to -10 V.

Supporting Information 7. SS and I_{on}/I_{off} ratio as a function of channel length

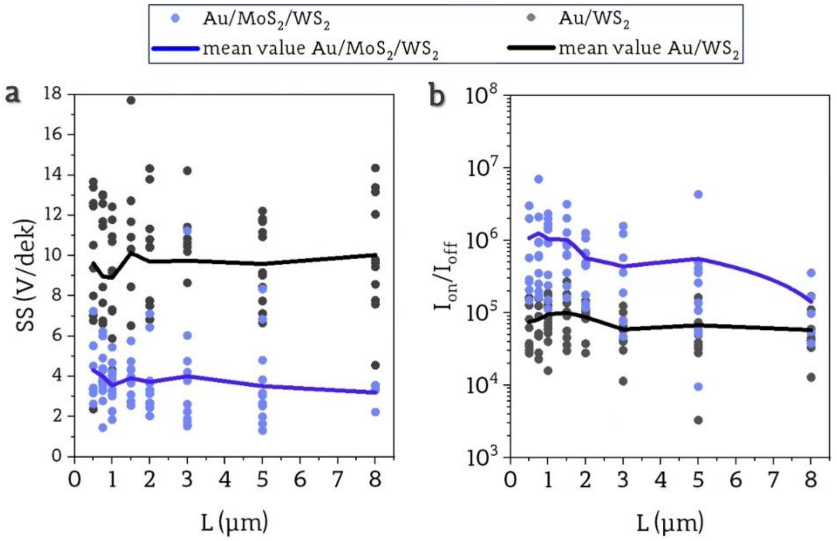

Figure S7 a) Subthreshold swing and b) I_{on}/I_{off} ratio as a function of channel length.

REFERENCES

(1) Saito, Y.; Kondo, T.; Ito, H.; Okada, M.; Shimizu, T.; Kubo, T.; Kitaura, R. Low Frequency Raman Study of Interlayer Couplings in WS $_2$ –MoS $_2$ van Der Waals Heterostructures. *Jpn. J. Appl. Phys.* **2020**, *59* (6), 062004. https://doi.org/10.35848/1347-4065/ab9400.

(2) Li, H.; Zhang, Q.; Yap, C. C. R.; Tay, B. K.; Edwin, T. H. T.; Olivier, A.; Baillargeat, D. From Bulk to Monolayer MoS $_2$: Evolution of Raman Scattering. *Adv Funct Materials* **2012**, *22* (7), 1385–1390. https://doi.org/10.1002/adfm.201102111.

(3) McCreary, K. M.; Hanbicki, A. T.; Singh, S.; Kawakami, R. K.; Jernigan, G. G.; Ishigami, M.; Ng, A.; Brintlinger, T. H.; Stroud, R. M.; Jonker, B. T. The Effect of Preparation Conditions on Raman and Photoluminescence of Monolayer WS2. *Sci Rep* **2016**, *6* (1), 35154. https://doi.org/10.1038/srep35154.

(4) Steinhoff, A.; Kim, J.-H.; Jahnke, F.; Rösner, M.; Kim, D.-S.; Lee, C.; Han, G. H.; Jeong, M. S.; Wehling, T. O.; Gies, C. Efficient Excitonic Photoluminescence in Direct and Indirect Band Gap Monolayer MoS $_2$. *Nano Lett.* **2015**, *15* (10), 6841–6847. https://doi.org/10.1021/acs.nanolett.5b02719.

(5) Zhang, J.; Du, L.; Feng, S.; Zhang, R.-W.; Cao, B.; Zou, C.; Chen, Y.; Liao, M.; Zhang, B.; Yang, S. A.; Zhang, G.; Yu, T. Enhancing and Controlling Valley Magnetic Response in MoS2/WS2 Heterostructures by All-Optical Route. *Nat Commun* **2019**, *10* (1), 4226. https://doi.org/10.1038/s41467-019-12128-2.

(6) Khan, M. F.; Ahmed, F.; Rehman, S.; Akhtar, I.; Rehman, M. A.; Shinde, P. A.; Khan, K.; Kim, D.; Eom, J.; Lipsanen, H.; Sun, Z. High Performance Complementary WS $_2$ Devices with Hybrid Gr/Ni Contacts. *Nanoscale* **2020**, *12* (41), 21280–21290. https://doi.org/10.1039/D0NR05737A.

(7) Park, W.; Kim, Y.; Jung, U.; Yang, J. H.; Cho, C.; Kim, Y. J.; Hasan, S. M. N.; Kim, H. G.; Lee, H. B. R.; Lee, B. H. Complementary Unipolar WS $_2$ Field-Effect Transistors Using Fermi-Level Depinning Layers. *Adv Elect Materials* **2016**, *2* (2), 1500278. https://doi.org/10.1002/aelm.201500278.
(8) Guimarães, M. H. D.; Gao, H.; Han, Y.; Kang, K.; Xie, S.; Kim, C.-J.; Muller, D. A.; Ralph, D. C.; Park, J. Atomically Thin Ohmic Edge Contacts Between Two-Dimensional Materials. *ACS Nano* **2016**, *10* (6), 6392–6399. https://doi.org/10.1021/acsnano.6b02879.
(9) Wang, Y.; Kim, J. C.; Wu, R. J.; Martinez, J.; Song, X.; Yang, J.; Zhao, F.; Mkhoyan, A.; Jeong, H. Y.; Chhowalla, M. Van Der Waals Contacts between Three-Dimensional Metals and Two-Dimensional Semiconductors. *Nature* **2019**, *568* (7750), 70–74. https://doi.org/10.1038/s41586-019-1052-3.
(10) Khalil, H. M. W.; Khan, M. F.; Eom, J.; Noh, H. Highly Stable and Tunable Chemical Doping of Multilayer WS $_2$ Field Effect Transistor: Reduction in Contact Resistance. *ACS Appl. Mater. Interfaces* **2015**, *7* (42), 23589–23596. https://doi.org/10.1021/acsami.5b06825.
(11) Sebastian, A.; Pendurthi, R.; Choudhury, T. H.; Redwing, J. M.; Das, S. Benchmarking Monolayer MoS2 and WS2 Field-Effect Transistors. *Nat Commun* **2021**, *12* (1), 693. https://doi.org/10.1038/s41467-020-20732-w.
